# *p*-Cresol Sulfate Is a Sensitive Urinary Marker of Fecal Microbiota Transplantation and Antibiotics Treatments in Human Patients and Mouse Models

**DOI:** 10.3390/ijms241914621

**Published:** 2023-09-27

**Authors:** Yuyin Zhou, Zheting Bi, Matthew J. Hamilton, Li Zhang, Rui Su, Michael J. Sadowsky, Sabita Roy, Alexander Khoruts, Chi Chen

**Affiliations:** 1 Department of Food Science and Nutrition, University of Minnesota, St. Paul, MN 55108, USA; zhou0882@umn.edu (Y.Z.); bixxx035@umn.edu (Z.B.); su000197@umn.edu (R.S.); 2 BioTechnology Institute, University of Minnesota, St. Paul, MN 55108, USA; hami0192@umn.edu (M.J.H.); sadowsky@umn.edu (M.J.S.); 3 Department of Surgery, University of Miami, Miami, FL 33136, USA; zhan1999@umn.edu (L.Z.); sabita.roy@miami.edu (S.R.); 4 Division of Gastroenterology, Department of Medicine, Center for Immunology, University of Minnesota, Minneapolis, MN 55455, USA; khoru001@umn.edu

**Keywords:** antibiotics, aromatic amino acid, *p*-cresol sulfate, fecal microbiota transplantation, gut microbiota, metabolomics, microbial metabolism

## Abstract

Fecal microbiota transplantation (FMT) has emerged as a highly effective therapy for recurrent *Clostridioides difficile* infection (rCDI) and also a potential therapy for other diseases associated with dysbiotic gut microbiota. Monitoring metabolic changes in biofluids and excreta is a noninvasive approach to identify the biomarkers of microbial recolonization and to understand the metabolic influences of FMT on the host. In this study, the pre-FMT and post FMT urine samples from 11 rCDI patients were compared through metabolomic analyses for FMT-induced metabolic changes. The results showed that *p*-cresol sulfate in urine, a microbial metabolite of tyrosine, was rapidly elevated by FMT and much more responsive than other microbial metabolites of aromatic amino acids (AAAs). Because patients were treated with vancomycin prior to FMT, the influence of vancomycin on the microbial metabolism of AAAs was examined in a mouse feeding trial, in which the decreases in *p*-cresol sulfate, phenylacetylglycine, and indoxyl sulfate in urine were accompanied with significant increases in their AAA precursors in feces. The inhibitory effects of antibiotics and the recovering effects of FMT on the microbial metabolism of AAAs were further validated in a mouse model of FMT. Overall, urinary *p*-cresol sulfate may function as a sensitive and convenient therapeutic indicator on the effectiveness of antibiotics and FMT for the desired manipulation of gut microbiota in human patients.

## 1. Introduction

Over the past decade, fecal microbiota transplantation (FMT) has become an effective treatment or explored as an alternative treatment for pathological conditions that are directly derived from or distantly associated with dysbiotic gut microbiota, including bowel diseases, hepatic encephalopathy, metabolic syndrome, neurodevelopmental and neurodegenerative disorders, and allergic diseases [1,2]. The efficacy of FMT is best highlighted by its 80–90% cure rate on recurrent *Clostridioides difficile* infection (rCDI), a medical condition that evades standard antibiotic therapies [3,4]. In rCDI, antibiotics actually make patients more vulnerable to *C. difficile* infection (CDI) by suppressing indigenous gut microbiota and their resistance against the colonization of enteric pathogens. The cycle of CDI recurrence develops, largely, due to the inability of antibiotic treatment to eradicate *C. difficile*, followed by worsening progressive dysbiosis with each round of treatment. For example, vancomycin-based antibiotic therapy was associated with ~20% of spontaneous recurrence of CDI after the first treatment [5,6], and the risk of recurrence increases by another 20% with each subsequent treatment [7]. In contrast, administration of fecal microbiota from healthy donors to rCDI patients results in rapid restoration of the microbial community structure in the colon and the resistance to rCDI [8].

The 700–1000 microbial species comprising the gut microbiota can be viewed as a major metabolic organ in the body [9]. Studies with germ-free mice have shown that up to 10% of the circulating metabolites are derivatives of microbial metabolism [10]. In rCDI patients, FMT leads to the rapid repair of decimated gut microbiota. Consequently, FMT in this clinical context is expected to alter the composition of microbiota-derived metabolites in the host. In fact, we, and others, have shown that FMT restores secondary bile acid metabolism in the large intestine, which further inhibits *C. difficile* spore germination and vegetative growth [5,11,12]. Therefore, examining FMT-associated metabolites could facilitate the mechanistic understanding of its pharmacodynamics, including the biochemical and physiologic effects that contribute to its efficacy, as well as potential side effects [13].

Urine provides a unique window to study the host metabolome as it is accumulative and rich in metabolites. Microbiota-derived metabolites in the urine are especially interesting because they are absorbed from the digestive tract and bioavailable to internal organs before excretion. However, the urinary metabolome has received little attention in FMT studies relative to FMT-induced changes in feces due to the challenges of identifying urinary microbial metabolites, as well as defining their associations with the changes in the gut microbiome.

In this study, FMT-induced changes of bioavailable microbial metabolites in urine samples of rCDI patients were examined by a liquid chromatography-mass spectrometry (LC-MS)-based metabolomic analysis of antibiotics and FMT treatments were subsequently performed in C57BL/6 mice to determine and validate the affected biogenesis pathways. Through this study, we aim to identify potential marker metabolites that may function as therapeutic indicators to monitor the effectiveness of antibiotics and FMT in the modification of gut microbiota.

## 2. Results

### 2.1. Analysis of FMT-Induced Metabolomic Changes in the Urine Samples of rCDI Patients

**Figure 1:**
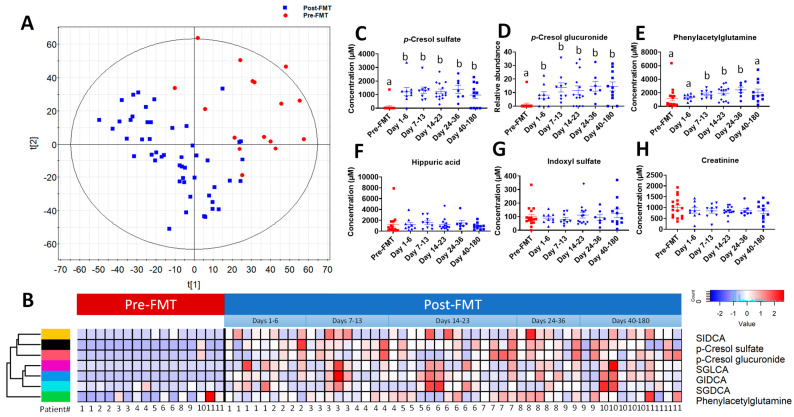
Identification of FMT-responsive urinary metabolites in rCDI patients. (**A**) the scores plot of a PLS-DA model on 16 pre-FMT and 50 post FMT urine samples from 11 rCDI patients. The t[1] and t[2] values represent the scores of each sample in the principal component 1 and 2, respectively. The urinary metabolites increased by FMT were subsequently identified and structurally defined (Figure S2 and Table S1). (**B**) heatmap and dendrogram of FMT-increased urinary metabolites from clustering analysis of urine samples collected from individual patients within defined time periods. Distribution of selected metabolites in pre-FMT and post FMT urine samples is presented in their concentrations or relative abundances. (**C**) *p*-cresol sulfate. (**D**) *p*-cresol glucuronide. (**E**) phenylacetylglutamine. (**F**) hippuric acid. (**G**) indoxyl sulfate. (**H**) creatinine. Relative abundances were presented as the ratios of the single ion counts of each metabolite versus the total ion counts of all detected metabolites. Statistical significances were calculated by one-way ANOVA followed by Tukey’s multiple comparisons, and different labels (a, b) indicate *p* < 0.05.

The distribution pattern of pre-FMT and post FMT urine samples in the unsupervised principal components analysis (PCA) model showed that the samples from the same subject tended to resemble each other, potentially reflecting the persistent influences of dietary practice and medications on the urinary metabolome of individual patients (Figure S1). However, in a supervised partial least squares-discriminant analysis (PLS-DA) model, the separation of pre-FMT and post FMT samples became clear (Figure 1A), indicating that FMT induced consistent metabolic changes in the urine of rCDI patients. Major urinary metabolites contributing to the classification of pre-FMT and post FMT samples were identified in the loadings S-plot of the orthogonal partial least-squares-discriminant analysis (OPLS-DA) model (Figure S2), and their chemical identities were confirmed by authentic standards and MSMS fragmentation as *p*-cresol sulfate, *p*-cresol glucuronide, phenylacetylglutamine, and the glycine conjugates of secondary bile acids and their sulfates, including glycoisodeoxycholate (GIDCA), sulfoglycodeoxycholate (SGDCA), sulfoglycolithocholate (SGLCA), and sulfoisodeoxycholate (SIDCA) (Table S1). The pattern and kinetics of FMT-induced changes in these urinary metabolites were further examined by a hierarchical cluster analysis (HCA) (Figure 1B) and quantitative analysis. While *p*-cresol sulfate was absent from 15 of 16 (93.7%) pre-FMT urine samples, its level was rapidly elevated within the first week of transplantation and sustained in the following months (Figure 1C). *p*-Cresol glucuronide shared a similar pattern with *p*-cresol sulfate (Figure 1D). Compared to two *p*-cresol conjugates, the FMT-induced increase in phenylacetylglutamine was more gradual and less dramatic, returning to its pre-FMT level on day 40–180 after the FMT (Figure 1E). The changes in secondary bile acid conjugates shared similar patterns with *p*-cresol conjugates and phenylacetylglutamine (Figure S3A–D). In contrast, hippuric acid and indoxyl sulfate, two microbial metabolites, as well as creatinine, a normal urine constituent, were unchanged by FMT (Figure 1F–H). These results indicated that *p*-cresol and selective secondary bile acids were the most FMT-responsive urinary metabolites in rCDI patients.

### 2.2. Tracing the Precursor Metabolites of p-Cresol Conjugates and Phenylacetylglutamine in the Fecal Samples of rCDI Patients

**Figure 2:**
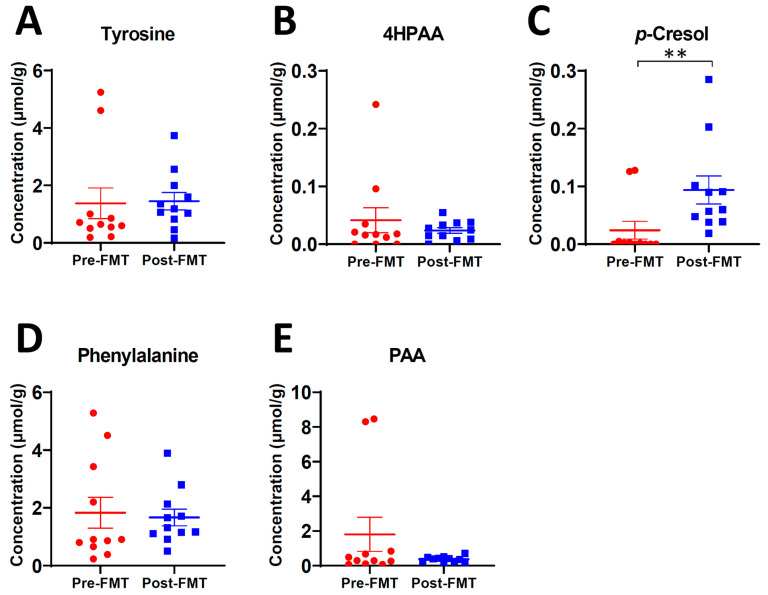
Concentrations of tyrosine, phenylalanine, and their microbial metabolites in the pre-FMT and post FMT fecal samples of 11 rCDI patients. The post FMT samples were collected on day 7 after FMT. (**A**) tyrosine. (**B**) 4HPAA. (**C**) *p*-cresol. (**D**) phenylalanine. (**E**) PAA. Statistical significances were calculated by paired two-tailed Student *t*-test (** *p* < 0.01). Moreover, 4HPPA and PPA, the two other microbial metabolites of tyrosine and phenylalanine, were not detected in these fecal samples.

To understand the causes behind the FMT-induced changes in *p*-cresol conjugates and phenylacetylglutamine in urine, the levels of their precursor metabolites in the pre-FMT and post FMT (day 7) fecal samples of rCDI patients were examined. The results showed that FMT significantly increased *p*-cresol in feces (Figure 2C) but did not affect tyrosine and 4-hydroxyphenylacetate (4HPAA), the two precursors of *p*-cresol (Figure 2A,B), as well as phenylalanine and phenylacetic acid (PAA) (Figure 2D,E). This observation indicated that the restoration of microbial *p*-cresol formation from tyrosine occurred within the first 7 days of FMT.

### 2.3. Analysis of Vancomycin-Responsive Metabolites in Mouse Urine and Feces

**Figure 3:**
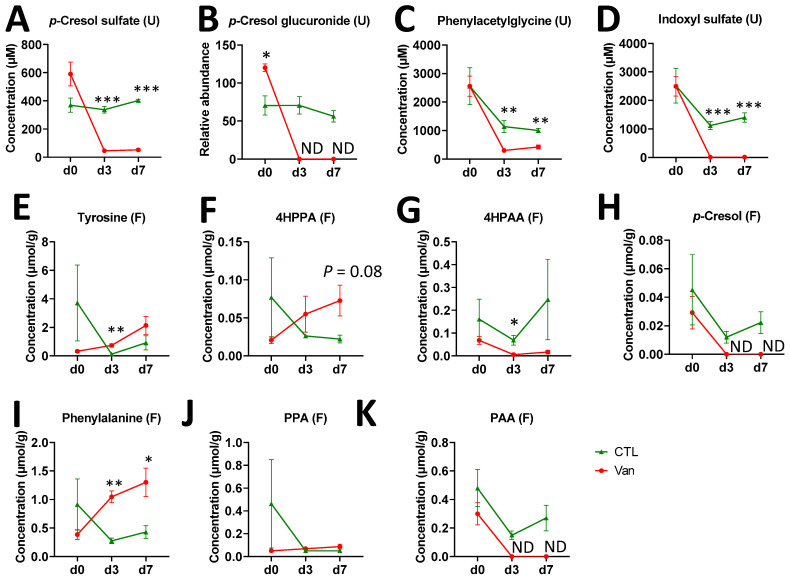
Influences of vancomycin on the microbial metabolites of tyrosine and phenylalanine in mouse urine (U) and feces (F). Urine and fecal samples were collected on day 0 (d0), 3 (d3), and 7 (d7) of control (CTL) and vancomycin (Van) treatments. (**A**) urinary *p*-cresol sulfate. (**B**) urinary *p*-cresol glucuronide. (**C**) urinary phenylacetylglycine. (**D**) urinary indoxyl sulfate. (**E**) fecal tyrosine. (**F**) fecal 4HPPA. (**G**) fecal 4HPAA. (**H**) fecal *p*-cresol. (**I**) fecal phenylalanine. (**J**) fecal PPA. (**K**) fecal PAA. Statistical significances were calculated by two-way ANOVA with multiple comparisons (* *p* < 0.05; ** *p* < 0.01; *** *p* < 0.001; ND, not detected). N = 4 per treatment per time point.

In our human FMT study, the rCDI patients took multiple rounds of oral vancomycin prior to FMT. To understand the effect of vancomycin on urine and fecal metabolome, vancomycin was orally administered to mice for 7 days. The PCA model on urinary metabolites showed a clear separation between control and vancomycin-treated samples on day 3 and 7 (Figure S4A), which was contributed to by a cluster of microbial metabolites, including *p*-cresol sulfate, *p*-cresol glucuronide, phenylacetylglycine, and indoxyl sulfate (Figure S4B). A quantitative analysis further confirmed that vancomycin treatment dramatically decreased these microbial metabolites in the urine (Figure 3A–D), which is similar to the low levels of *p*-cresol conjugates and phenylacetylglutamine in the pre-FMT rCDI patients. The influences of vancomycin on *p*-cresol and PAA biogenesis were further examined by measuring the metabolites of tyrosine → *p*-cresol and phenylalanine → PAA pathways in mouse feces. The results showed that vancomycin significantly elevated the concentrations of tyrosine and phenylalanine (Figure 3E,I) and did not have a significant influence on 4-hydroxyphenylpropionic acid (4HPPA) and phenylpropionic acid (PPA), the respective transamination metabolites of tyrosine and phenylalanine (Figure 3F,J). In contrast, vancomycin abolished the presence of 4HPAA, *p*-cresol, and PAA in mouse feces on both day 3 and day 7 of the treatment (Figure 3G,H,K).

The changes in tyrosine, phenylalanine, and their microbial metabolites were a part of vancomycin-induced changes in the mouse fecal metabolome, as shown by the separation of control and vancomycin-treated samples in the PCA model (Figure S4C). Fecal bile acids and amino acids were also greatly affected by vancomycin in mice. Vancomycin increased taurine-conjugated primary bile acids and their sulfates, including taurocholic acid (TCA), tauromuricholic acid (TMCA), sulfotaurocholic acid (STCA), and sulfotaurochenodeoxycholic acid (STCDCA), while decreased secondary bile acids, including deoxycholic acid (DCA), muricholic acid (MCA), isodeoxycholate (IDCA), ursodeoxycholic acid (UDCA), and lithocholic acid (LCA) (Figure S4D). Besides tyrosine and phenylalanine, other free amino acids in feces were also increased by vancomycin (Figure S4D). The accumulation of amino acids and primary bile salts in vancomycin-treated mice was presumably due to the deprivation of their microbial metabolism.

### 2.4. Analysis of FMT-Induced Metabolomic Changes in the Colon Digesta Samples of Mice

**Figure 4:**
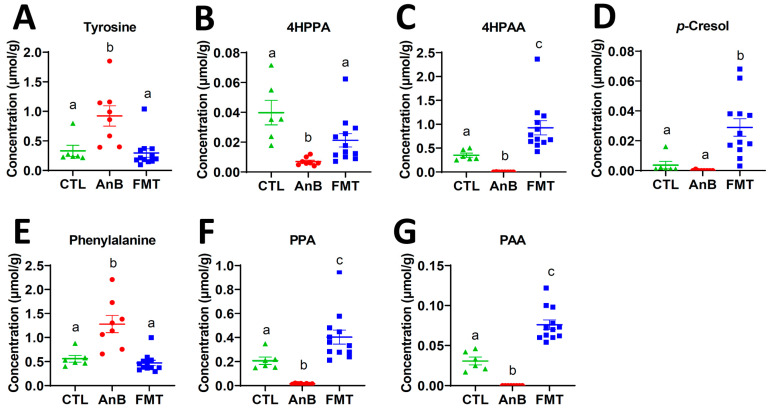
Concentrations of tyrosine, phenylalanine and their metabolites in the colon digesta samples of mice receiving antibiotics (AnB) and FMT. (**A**) tyrosine. (**B**) 4HPPA. (**C**) 4HPAA. (**D**) *p*-cresol. (**E**) phenylalanine. (**F**) PPA. (**G**) PAA. Statistical significances were calculated by one-way ANOVA followed by Tukey’s multiple comparisons, and different labels (a, b, c) indicate *p* < 0.05. N = 6 in control (CTL) group; n = 8 in AnB group; n = 12 in FMT group.

To further investigate the sensitivity of microbial *p*-cresol biogenesis to antibiotics and FMT, FMT was performed after the treatment of a broad-spectrum antibiotic cocktail in mice. The metabolomic analysis of the colonic digesta showed the clear separation of the antibiotics group from the control and the FMT groups in the PCA model (Figure S5A), indicating antibiotics triggered disruption of the colon digesta metabolome and FMT induced recovery. The separation of the three treatment groups was contributed to by the higher abundances of free amino acids and primary bile salts (TCA and TMCA) in the antibiotics group and the higher abundances of secondary bile acids (MCA and DCA), as well as 4HPAA and *p*-cresol, in the control and FMT groups (Figure S5B). A quantitative analysis of the metabolites in the tyrosine → *p*-cresol and phenylalanine → PAA biogenesis pathways showed that tyrosine and phenylalanine were elevated by antibiotic treatment, while FMT reduced their concentrations back to the control (Figure 4A,E). The concentrations of 4HPPA, 4HPAA, PPA, and PAA in the colon digesta were decreased by antibiotics and then increased by FMT (Figure 4B,C,F,G). The concentration of *p*-cresol in the FMT samples was greater than that of the control and antibiotics groups (Figure 4D).

## 3. Discussion

**Figure 5:**
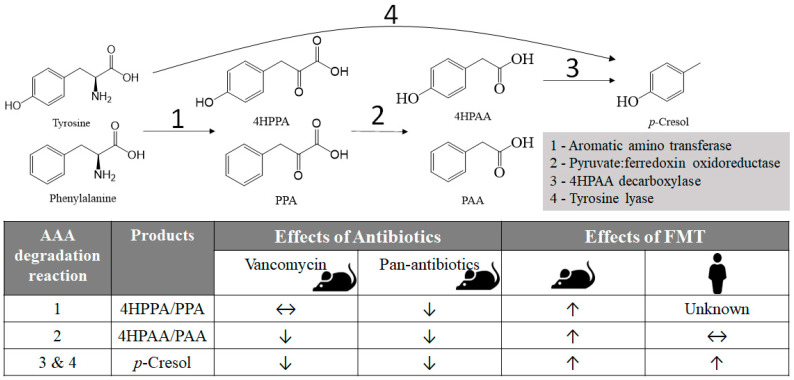
Summary on the observed effects of FMT and antibiotics on microbial metabolism of tyrosine and phenylalanine to form *p*-cresol and phenylacetate (PAA), which are the respective precursors of *p*-cresol conjugates and phenylacetylglutamine, in human and mouse urine. Enzymes (1–4) responsible for the formation of intermediates and end products of AAA degradation reactions are annotated. The effects of FMT and antibiotics are shown as ↑ (increased), ↓ (decreased), or ↔ (not affected).

The metabolic reactions responsible for the formation of microbial AAA metabolites have been well characterized. As an end product of tyrosine degradation, *p*-cresol can be produced directly through a one-step reaction catalyzed by tyrosine lyase [14], but more commonly through a cascade that is mediated first by aromatic amino transferase to yield 4HPPA, followed by the formation of 4HPAA by pyruvate: ferredoxin oxidoreductase and then the production of *p*-cresol by 4HPAA decarboxylase [15] (Figure 5). The same transamination and oxidation reactions in this cascade are also responsible for converting phenylalanine to PPA and then PAA (Figure 5). Endogenous metabolism further metabolizes absorbed *p*-cresol and PAA to *p*-cresol conjugates and phenylacetylglutamine, respectively, for urinary excretion [16]. In this study, the identification of microbial AAA metabolites, especially *p*-cresol and its conjugates, as the most responsive metabolites to antibiotics and FMT highlights the sensitivity of microbial AAA metabolism to the changes of gut microbiota. The causes of these microbial interventions induced metabolic events and their potential applications for monitoring FMT and pathological conditions are discussed as follows.

### 3.1. Sensitivity of p-Cresol and PAA Biogenesis to Microbial and Chemical Interventions

Both humans and mice, the two subjects examined in this study, produce similar microbial metabolites of AAAs by hosting comparable AAA-degrading microbiota in their intestinal tracts [15,17,18]. However, microbial metabolism of individual AAAs may differ in the sensitivity to microbial and chemical interventions across species (human and mouse) and metabolizing reactions. For the rCDI patients in this study, FMT increased the conversions of tyrosine and phenylalanine to urinary *p*-cresol conjugates and phenylacetylglutamine, respectively, but did not affect urinary indoxyl sulfate, a microbial metabolite of tryptophan [17], and hippuric acid, a microbial metabolite of aromatic compounds [19], as well as creatinine, a normal urine constituent. Different sensitivities of individual AAA degradation reactions to FMT were further revealed by profiling *p*-cresol, PAA, and their precursors in rCDI patients, showing the increase in fecal *p*-cresol but not others on day 7 of FMT, a profile resembling the status of urinary *p*-cresol conjugates and phenylacetylglutamine in the first week of FMT (Figure 1 and Figure 2). This observation indicated that FMT might quickly promote the decarboxylation and lysis reactions that directly produce *p*-cresol but have little effect on the transamination and oxidation reactions for producing 4HPPA and PAA in the early phase of FMT (Figure 5). In two mouse experiments, vancomycin increased tyrosine and phenylalanine and diminished PAA, 4HPAA, and *p*-cresol with no influences on 4HPPA and PPA in feces, while pan-antibiotics elevated tyrosine and phenylalanine and depleted all other metabolites in the colon digesta (Figure 3 and Figure 4). The difference between pan-antibiotics and vancomycin-alone treatments further confirmed that individual reactions in *p*-cresol and PAA biogenesis pathways and the bacteria carrying these reactions had different sensitivities to the inhibitory effects of antibiotics. The sensitivity of *p*-cresol biogenesis was also observed in other forms of microbial interventions, such as pre- and probiotic treatments. It has been shown that the supplementation of *Lactobacillus* strains decreased the excretion of urinary *p*-cresol sulfate in healthy women [20]. Besides microbial interventions, chemical intervention of *p*-cresol biogenesis could also be achieved as shown in our recent studies, in which microbial AAA degradation, including 4HPAA decarboxylase-catalyzed *p*-cresol production, was competitively inhibited by green tea polyphenol intake in humans [21] and rutin feeding in dairy cows [22]. Therefore, profiling *p*-cresol and other AAA degradation products in urine and feces can serve as an effective analytical approach to determine the status of microbial AAA metabolism and its sensitivity to microbial and chemical interventions.

The sensitivity of *p*-cresol biogenesis to interventions has clear practical implications. In clinical practice, 10–40% of FMT treatments fail to cure rCDI [23]. The poor engraftment of donor bacteria likely accounts for a significant fraction of these treatment failures [24]. However, symptoms of CDI relapse generally occur in the second or third weeks after FMT [25]. It is possible that measurement of *p*-cresol in stool or its derivatives in urine can be developed as an early indicator of the successful engraftment of donor bacteria before regrowth of *C. difficile*, allowing for the early re-treatment of patients destined to develop symptoms of recurrent CDI infection.

### 3.2. Associations among p-Cresol, C. difficile, and FMT

*C. difficile* is known to produce *p*-cresol and also be resistant to the bacteriostatic activity of *p*-cresol [26,27]. Hence, *p*-cresol has been designated as a marker for CDI identification [28,29]. In this study, the absence of *p*-cresol in pre-FMT patients reflects the absence of metabolically active *C. difficile* from effective antibiotic treatment. However, the rapid and persistent presence of *p*-cresol in our post FMT patients were correlated to the effective recovery of functional and rCDI-resistant gut microbiota, rather than the recurrence of CDI. In fact, Farowski et al. also reported an increase in urinary *p*-cresol sulfate in CDI patients following FMT [30]. Therefore, *p*-cresol should no longer be considered as a specific metabolite marker of *C. difficile* and CDI. This conclusion is supported by the distribution of 4HPAA decarboxylase, a glycyl radical enzyme responsible for converting 4HPAA to *p*-cresol, in gut microbes as it was not only purified and cloned from *C. difficile*, but also from other related but nonpathogenic species [31]. Moreover, diverse anaerobic bacterial species belonging to the genera of *Faecalibacterium*, *Eubacterium*, *Anaerostipes*, *Ruminococcus*, *Bacteroides*, *Bifidobacterium*, and *Coriobacteriaceae* host the genes of other tyrosine degradation enzymes and are capable of producing *p*-cresol [14]. Many of them are constitutive members of human gut microbiota. Therefore, FMT quickly introduced these *p*-cresol producers to colonize the gut, leading to a rapid restoration of *p*-cresol synthesis and the urinary excretion of *p*-cresol sulfate.

Another feature of the FMT-induced presence of *p*-cresol sulfate was its coincidence with the elevation of urinary secondary bile acid conjugates in rCDI patients. Our previous fecal metabolomic analysis has shown that the microbial metabolism of bile acids was abolished by vancomycin and then rapidly recovered by FMT in the same rCDI patients [5]. We further proved that the rapid recovery of secondary bile acid synthesis by FMT inhibited the germination and growth of *C. difficile* and then prevented rCDI [11]. Our fecal microbiomic analysis has revealed that the bile acid-metabolizing bacteria, including the *Lachnospiraceae*, *Ruminococcaceae*, and *Bacteroidaceae* species, comprised 54% and 57% of total operational taxonomic units (OTU) in FMT donors and post FMT rCDI patients, respectively, but were largely absent in the pre-FMT rCDI patients after vancomycin treatment [5]. Interestingly, many *Lachnospiraceae*, *Ruminococcaceae*, and *Bacteroidaceae* species also produce *p*-cresol (summarized in Table S2) [14,32]. Therefore, the simultaneous increases in urinary *p*-cresol sulfate and secondary bile acid conjugates in post FMT rCDI patients further justify the value of *p*-cresol sulfate as an efficacy marker of FMT.

### 3.3. Challenges in Understanding the Potential Consequences of Altered p-Cresol Biogenesis

Interpreting the biological significances of altered *p*-cresol biogenesis is confounded by the associations of *p*-cresol with both beneficial and adverse effects on related health events. As stated above, *C. difficile* produces *p*-cresol [27]. However, restored *p*-cresol biogenesis by FMT was also coincident with the resistance to rCDI in this study. For chronic kidney disease (CKD) patients, *p*-cresol sulfate, which is hard to be removed by dialysis, is regarded as a uremic toxin for its potential contribution to renal and cardiovascular damages [32], as well as to insulin resistance [33]. On the other hand, the serum *p*-cresol level was negatively correlated with type II diabetes in humans, and chronic exposure to a non-toxic level of *p*-cresol reduced adiposity, glucose intolerance, and liver fat in mice [34]. As for neurological disorders, elevated urinary *p*-cresol sulfate was observed in autistic children [35], and *p*-cresol was shown to alter brain dopamine metabolism and promote autistic behaviors in mice [36]. On the other hand, a low-dose exposure of *p*-cresol might have neuroprotective effects by inducing the secretion of brain-derived neurotrophic factors and promoting neuronal cell structural remodeling [37]. All these observations warrant more investigations on the bioactivities of *p*-cresol, especially dose-dependent responses.

### 3.4. Limitation of Observed Changes in p-Cresol Biogenesis after Antibiotics and FMT Treatments

Due to the challenges of collecting sampling in human clinical care and the retrospective nature of this study, urine samples prior to vancomycin treatment in the rCDI patients were not collected. Therefore, the status of *p*-cresol sulfate in their pre-treatment urine metabolome was unknown. However, since *p*-cresol sulfate was widely accepted as a constitutive metabolite in human urine from the fermentation of undigested proteins in both vegetarians and omnivores [38], its presence in the pre-vancomycin patients would be expected. Furthermore, no placebo controls of antibiotics and FMT treatments were included in our clinical study as all patients were treated with vancomycin following our established clinical procedure [5]. Nevertheless, a separate randomized controlled trial in rCDI patients showed that the microbiome recovery in the absence of FMT took months after vancomycin treatment [39], which may also imply a slow recovery of *p*-cresol biogenesis. As for the current study, the associations of *p*-cresol biogenesis in human patients with antibiotics and FMT were only validated by two mouse experiments. Therefore, additional human studies with larger patient cohorts and placebo controls are needed to confirm and consolidate these conclusions.

## 4. Materials and Methods

### 4.1. Patients, FMT Procedure, and Sample Collection

The details on the cohort of CDI patients and the procedure of FMT have been described previously [5,40]. The procedure and the sample collection were approved by the Institutional Review Board (IRB) at the University of Minnesota (IRB approval number 0901M56962). The clinical demographics of the patients, their clinical course, and changes in their stool microbiome and metabolomics were described previously [5]. Briefly, all 11 patients had rCDI syndrome after multiple rounds of antibiotic treatments failed to resolve the infection. Prior to FMT procedure, all patients took 125 mg of vancomycin orally four times per day until 2 days prior to FMT procedure. After receiving the fecal microbiota from one of two healthy donors via colonoscopy infusion, all patients achieved clinical recovery of intestinal functions (≤3 bowel movements per day and normalization of stool consistency) and clinical cure of rCDI (no relapse of the infection over two-month period of follow-up after the FMT). A total of 16 morning urine samples were collected prior to the FMT and 50 urine samples between day 0 and day 180 after the FMT. Fecal samples were collected prior to FMT and on day 7 following FMT. Samples were stored at −80 °C until processing.

### 4.2. Mouse Vancomycin Treatment

Eight-week-old male C57BL/6 mice were acquired from Charles River Lab (Wilmington, MA, USA). All mice were housed in the University of Minnesota animal facility at a constant temperature of 21 °C under a 12 h light/dark cycle and had access to water and feed ad libitum. Treatment procedures were approved by the University of Minnesota Institutional Animal Care and Use Committee. After acclimation, mice were randomly assigned to two groups (n = 4 per group) and were given either 100 mg/kg vancomycin HCl aqueous solution or water through oral gavage every 12 h for 7 days following an established dosing formula [41]. Urine and feces from each animal were collected using metabolic cage on days 3 and 7 of the treatment. All samples were stored at −80 °C until processing.

### 4.3. Mouse FMT

FMT was conducted on mice based on our previous protocol [42]. All procedures were approved by the Institutional Animal Care and Use Committee of the University of Miami. Briefly, 26 C57BL/6 mice were housed in pathogen-free (SPF) conditions and maintained on a 12 h light/dark cycle at 21 °C with ad libitum access to food and water. Antibiotic cocktail (0.5 mg/mL bacitracin, 2 mg/mL neomycin, 0.2 mg/mL vancomycin and 1.2 µg/mL pimaricin) was freshly prepared every day in drinking water and was given to 20 mice for 7 days prior to FMT. Fecal suspension for FMT was prepared using the pooled feces from 10 donor mice. Briefly, 200 mg of pooled feces was suspended in 1 mL sterile PBS, filtered through 70 µm cell strainer, and centrifuged at 6000× *g* for 20 min to obtain bacteria pellet. About 10^10^ CFU/mL fecal bacteria was suspended in 6% NaHCO_3_ buffer with 20% sucrose. Starting on day 8, 12 mice were orally gavaged with 200 µL of freshly prepared fecal suspension for 7 consecutive days before sacrifice (FMT group). The other 8 mice continued receiving the same pan-antibiotics cocktail for 7 consecutive days before sacrifice (AnB group). Another 6 mice received drinking water throughout the experiment (CTL group). Colon digesta were collected at sacrifice and stored immediately at −80 °C until further analysis.

### 4.4. Reagents

All solvents, chemical reagents, and standards were purchased form Sigma-Aldrich (St. Louis, MO, USA) unless stated otherwise. LC-MS grade acetonitrile was purchased from Fisher Scientific (Pittsburgh, PA, USA). *p*-Cresol sulfate was purchased from Cayman Chemicals (Ann Arbor, MI, USA). Cholic acid and diethyl phosphorocyanidate (DEPC) were purchased from Alfa Aesar (Ward Hill, MA, USA). Isodeoxycholic acid was purchased from Steraloids (Newport, RI, USA).

### 4.5. Synthesis of Metabolite Standards for Structural Confirmation

Sulfated standards, including sulfoisodeoxycholate, sulfoglycodeoxycholate, sulfoglycolithocholate, were synthesized by microscale reactions in which chlorosulfonic acid was added to the ice-cooled solutions of *p*-cresol, cholesterol, isodeoxycholic acid, glycolithocholic acid, and glycodeoxycholic acid in dichloromethane, respectively [43]. The mixture was stirred at 4 °C for 10 min. After solvent evaporation, the products were reconstituted in 50% aqueous acetonitrile and then neutralized by 2N NaOH prior to the LC-MS analysis. To synthesize glycoisodeoxycholate, glycine, DEPC, and triethylamine were mixed with a solution of isodeoxycholic acid in dimethylfuran [44]. The mixture was stirred at room temperature for 30 min. After solvent evaporation, the product was reconstituted in 50% aqueous acetonitrile prior to the LC-MS analysis.

### 4.6. LC-MS Analysis of Biological Sample Extracts

*Preparation of urine, feces, and colon digesta extracts for LC-MS analysis.* The human urine sample was mixed with an equal volume of 50% acetonitrile. The mouse urine was mixed with 19 volumes of 50% acetonitrile. The mixture was centrifuged at 18,000× *g* for 10 min to remove particles and precipitates. Feces and colon digesta samples were suspended in ten volumes (*v*/*w*) of 50% acetonitrile and extracted by vortexing and sonication for 10 min. The suspension was centrifuged at 18,000× *g* for 10 min twice to separate supernatant and precipitate. The supernatant was collected for LC-MS analysis. For detection of metabolites containing amino groups in their structure, the samples were derivatized with dansyl chloride (DC) prior to the LC-MS analysis.

*Sample derivatization.* For the detection of metabolites containing amino groups or hydroxyl groups in their structure, the samples were derivatized with dansyl chloride (DC) prior to the LC-MS analysis. Briefly, 5 μL of samples or standards were mixed with 5 μL of 100 µM *d*_5_-tryptophan (internal standard), 50 μL of 10 mM sodium carbonate, and 100 μL of DC (3 mg/mL in acetone). The mixture was incubated at 60 °C for 15 min and centrifuged (18,000× *g*) for 10 min, the supernatant was transferred into a HPLC vial for LC-MS analysis.

*Conditions of LC-MS analysis.* For LC-MS analysis, a 5 µL aliquot of sample extract was injected into an Acquity^TM^ UPLC system (Waters, Milford, MA, USA) and separated on a BEH C18 column by a gradient of mobile phase over a 10 min run (Table S3). The LC eluent was introduced into a SYNAPT QTOF mass spectrometer (Waters) for accurate mass measurement and ion counting (Table S3). For accurate mass measurement, the mass spectrometer was calibrated with sodium formate solution (range *m*/*z* 50–1000) and monitored by real-time intermittent injection of the lock mass leucine enkephalin ([M+H]^+^ = 556.2771 *m*/*z* and ([M−H]^−^ = 554.2615 *m*/*z*). Additional structural information was obtained by tandem MS (MS/MS) fragmentation with collision energies ranging from 15 to 30 eV.

*Quantitative Analysis*. The concentrations of targeted metabolites were determined by calculating the ratio between their individual peak areas and the peak area of internal standard and fitting with a standard curve using QuanLynx^TM^ software version 4.2 (Waters, Milford, MA), and compared with the values of respective limits of quantitation (LOQ). For 4HPAA, *p*-cresol, and PAA quantification, the samples with concentrations less than the LOQ were substituted with half LOQ for data visualization and statistical analysis.

### 4.7. Multivariate Analysis and Biomarker Identification

Data generated in different LC-MS analyses were combined for each sample matrix. Chromatographic and mass spectral data were deconvoluted by using MarkerLynx^TM^ software version 4.2 (Waters, Milford, MA, USA). A multivariate data matrix comprising sample identity, ion identity (retention time, RT, and *m*/*z*) and relative ion abundance was generated through centroiding, deisotoping, filtering, peak recognition, and integration, and was further exported into SIMCA-P+^TM^ software version 12 (Umetrics, Kinnelon, NJ, USA) for multivariate data analysis [45]. Unsupervised principal components analysis (PCA), together with supervised projection to latent structures-discriminant analysis (PLS-DA) and orthogonal partial least-squares-discriminant analysis (OPLS-DA), were adopted to analyze the data of the samples. Major latent variables in the data matrix were described in a scores scatter plot of established multivariate model. Metabolites affected by antibiotics and FMT were identified by analyzing ions contributing to the separation of samples from different treatment groups in the multivariate models [45,46]. The chemical identities of metabolite markers were determined by accurate mass measurement, elemental composition analysis, database searches (Human Metabolome Database: http://www.hmdb.ca/) accessed on 5 May 2023, MS/MS fragmentation, and comparisons with authentic standards. The heatmaps of metabolite markers from hierarchical cluster analysis were created using the heatmap.2 function in the gplots tools of R package (https://cran.r-project.org/, accessed on 5 May 2023).

### 4.8. Statistics

Experimental values are expressed as mean ± standard error of the mean (SEM). After being tested for normality and transformed using Log_2_ if needed, the data were statistically analyzed by paired two-tailed Student *t*-test (human FMT fecal data), one-way ANOVA followed by Tukey’s multiple comparisons (human FMT urine data and mouse FMT data), or two-way ANOVA with multiple comparisons (vancomycin-treated mouse data) using GraphPad Prism 5 (GraphPad Software, Inc., La Jolla, CA, USA). A *p* value of <0.05 was considered as statistically significant.

## 5. Conclusions

Our metabolomic examination of rCDI patients and mouse models revealed that *p*-cresol biogenesis is more responsive to antibiotics and FMT treatments than other microbial degradation pathways of AAAs, making urinary *p*-cresol sulfate a sensitive metabolite marker for monitoring the effectiveness of antibiotics and FMT treatments for the desired manipulation of gut microbiota in human patients.

## Data Availability

The processed data are contained within the article and Supplementary Materials. The raw data of chromatographic and spectrometric analyses can be requested from the corresponding authors.

## Supplementary Materials

The following supporting information can be downloaded at: https://www.mdpi.com/article/10.3390/ijms241914621/s1. References [47,48,49,50,51] are cited in the Supplementary Materials.
