# Supplementary material for: p-Cresol Sulfate Is a Sensitive Urinary Marker of Fecal Microbiota Transplantation and Antibiotics Treatments in Human Patients and Mouse Models
Source: Int J Mol Sci. 2023 Sep 27;24(19):14621. doi: 10.3390/ijms241914621 (PMC10572327; doi:10.3390/ijms241914621)
Supplement: Supplementary file 1 [file ijms-24-14621-s001.zip › ijms-2521569-supplementary.pdf]

SUPPLEMENTARY DATA

Table S1. Urinary metabolite markers of FMT in R-CDI patients

Ions	RT (min)	*m*/*z* of $[M - H]^-$	Formula	Identity
I_u	2.56	187.0065	$C_7H_8O_4S$	*p*-Cresol sulfate
I_u'	2.56	107.0497	C_7H_8O	*p*-Cresol sulfate in source fragment
I_u''	2.56	79.9568	HSO_3	*p*-Cresol sulfate in source fragment
II_u	5.17	448.3068	$C_{26}H_{43}NO_5$	Glycoisodeoxycholate (GIDC)
III_u	4.94	528.2622	$C_{26}H_{43}NO_8S$	Sulfoglycodeoxycholate (SGDCA)
IV_u	5.12	512.2696	$C_{26}H_{43}NO_7S$	Sulfoglycolithocholate (SGLCA)
V_u	4.69	471.2412	$C_{24}H_{40}O_7S$	Sulfoisodeoxycholate (SIDCA)
VI_u	1.76	283.0818	$C_{13}H_{16}O_7$	*p*-Cresol glucuronide
VII_u	1.77	263.1032	$C_{13}H_{16}N_2O_4$	Phenylacetylglutamine

Table S2. Significantly changed bacterial in patient and donor samples and their ability to form secondary bile acids and *p*-cresol*

Average No. of OTUs [5]			*P* (pre- vs. post-FMT) [5]	Family	Strains [47]	Secondary bile acid producer	*p*-Cresol producer [14]
Donor	Pre-FMT	Post-FMT					
4,002	286	4,737	<0.0001	Lachnospiraceae	*C. aerotolearans*	Unknown	Unknown
					C. aminophilum	Unknown	No
					C. aminovalericum	Unknown	Yes
					C. celerecrescens	Unknown	Yes
					C. clostridioforme	Unknown	Yes
					C. coccoides	Unknown	Yes
					C. nexile	Unknown	Yes
					C. oroticum	Unknown	Yes
					C. saccharolyticum	Unknown	Yes
					C. populeti	Yes [12]	Unknown
					C. symbiosum	Unknown	Yes
					C.sphenoides	Unknown	Yes
					C. xylanolyticum	Unknown	Unknown
					C. scindens	Yes [5]	Yes
					C. hylemonae	Yes [48]	Yes
					Acetitomaculum ruminis	Unknown	Unknown
					Coprococcus eutatus	Unknown	No
					Eubacterium cellulosolvens	Unknown	No
					Peptostreptococcus productus	Unknown	Unknown
					Roseburia cecicola	Unknown	Unknown
					Ruminococcus torques	Unknown	Yes
					Streptococcus hansenii	Unknown	No
1,749	112	3,797	0.0002	Ruminococcaceae	*C. leptum*	Yes [49]	Yes
					C. sporosphaeroides	Unknown	Unknown
					C. cellulosi	Unknown	Unknown
					F. prausnitzii	Yes [50]	No
6451	25	4348	0.0009	Bacteroidaceae	*B. uniformis*	Yes [51]	Yes

*Details of the citations in this table are presented in References of the article.

Table S3. LC-MS Data Acquisition Conditions in a 10-minute Run

Target compounds	Column type	Mobile phase	MS detection mode	Capillary and cone voltage	Source and desolvation temperature	Cone and desolvation gas	Collision gas
Urinary metabolites	BEH C18	A: H_2O containing 10 nM NH_4OAc, pH9<br>B: H_2O/ACN = 5:95 (v/v) containing 10 nM NH_4OAc, pH9	negative	0.2 kV, 40 V	120 °C, 350 °C	50 L/h, 600 L/h (N_2)	Argon
Amino acids and metabolites	BEH C18	A: H_2O containing 0.1% formic acid (v/v)<br>B: Acetonitrile containing 0.1% formic acid (v/v)	Positive	0.2 kV, 40 V	120 °C, 350 °C	50 L/h, 600 L/h (N_2)	Argon

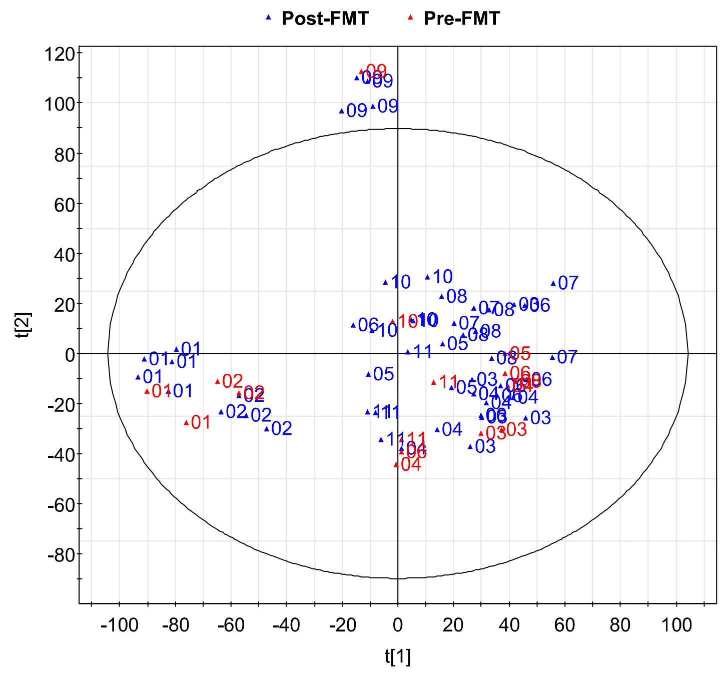

Figure S1. The scores plot of a PCA model on pre-FMT and post-FMT urine samples. The *t*[1] and *t*[2] values represent the scores of each sample in the principal component 1 and 2, respectively. The samples from 11 rCDI patients are labeled from 01 to 11.

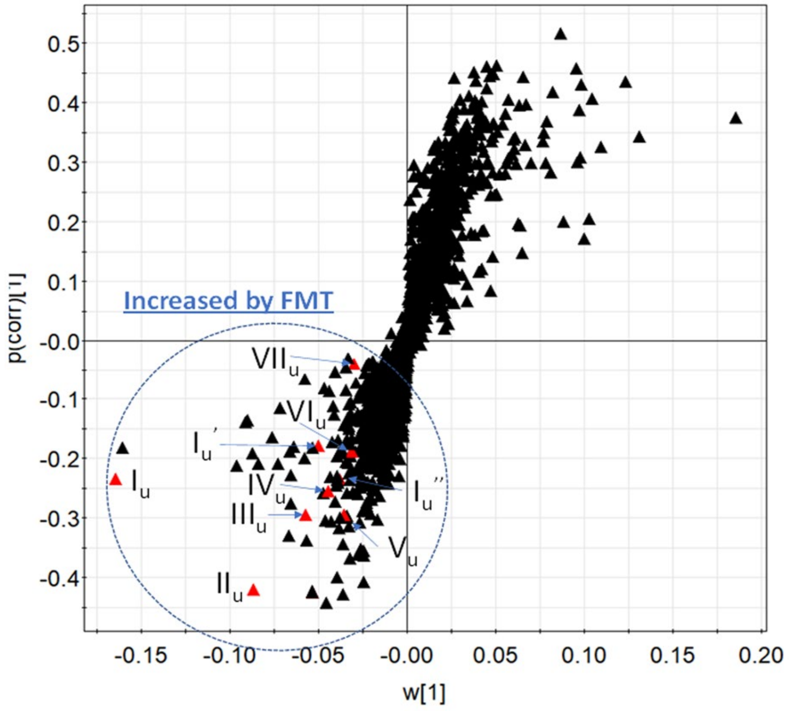

Figure S2. The S-plot of OPLS-DA model on pre-FMT and post-FMT urine samples. Major urinary metabolites increased by FMT (I_u –VII_u) were labeled and their chemical identities are enlisted in Table S1.

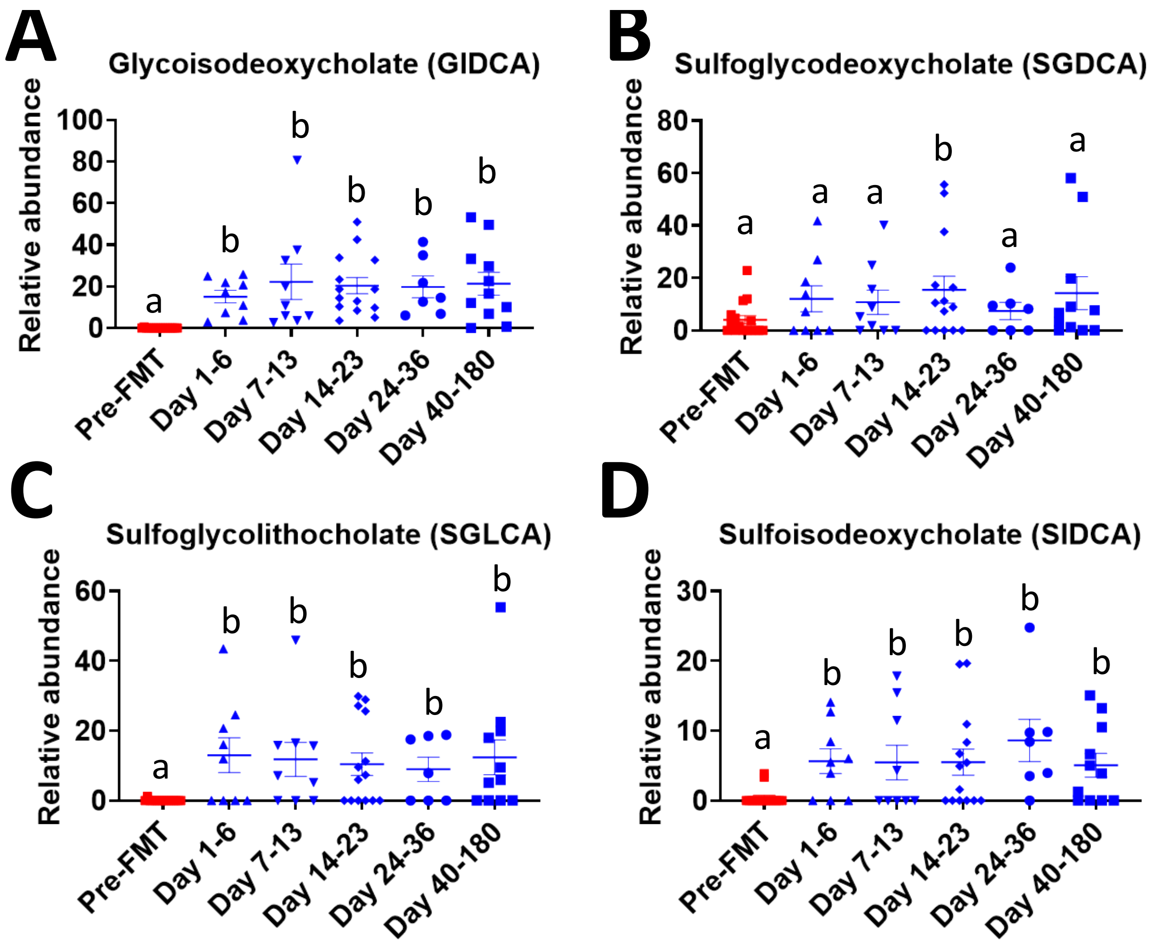

Figure S3. Distribution of FMT-responsive secondary bile acids in pre-FMT and post-FMT urine samples. (A) Glycoisodeoxycholate. (B) Sulfoglycodeoxycholate. (C) Sulfoglycolithocholate. (D) Sulfoisodeoxycholate. Relative abundances were presented as the ratios of the single ion counts of each metabolite versus the total ion counts of all detected metabolites. Statistical significances were calculated by one-way ANOVA followed by Tukey's multiple comparisons, and different labels (a, b) indicate $P < 0.05$.

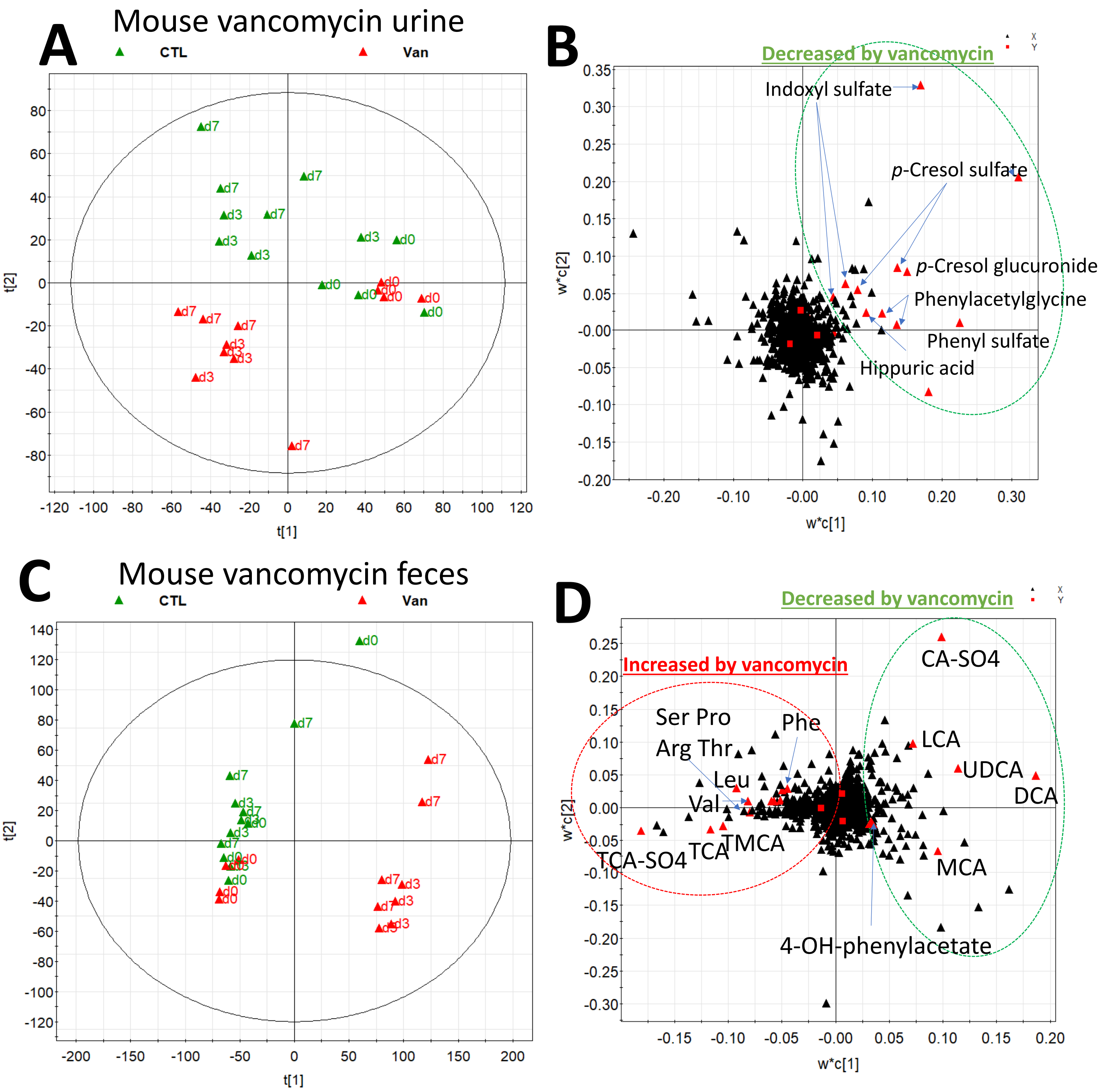

Figure S4. Identification and characterization of vancomycin-responsive urinary and fecal metabolites in mice. Urine and fecal samples were collected on day 0, 3, and 7 of control (water) and vancomycin treatments. (A) The scores plot of a PCA model on the urine samples collected on day 0 (d0), day 3 (d3), and day 7 (d7) of control (water) and vancomycin treatments. (B) The loadings plot of the PCA model on urine samples. Major microbial metabolites decreased by vancomycin are labeled. (C) The scores plot of a PCA model on the fecal samples collected on day 0 (d0), day 3 (d3), and day 7 (d7) of control (water) and vancomycin treatments. (D) The loadings plot of the PCA model on fecal samples. Major vancomycin-responsive metabolites, including amino acids and bile acids, are labeled.

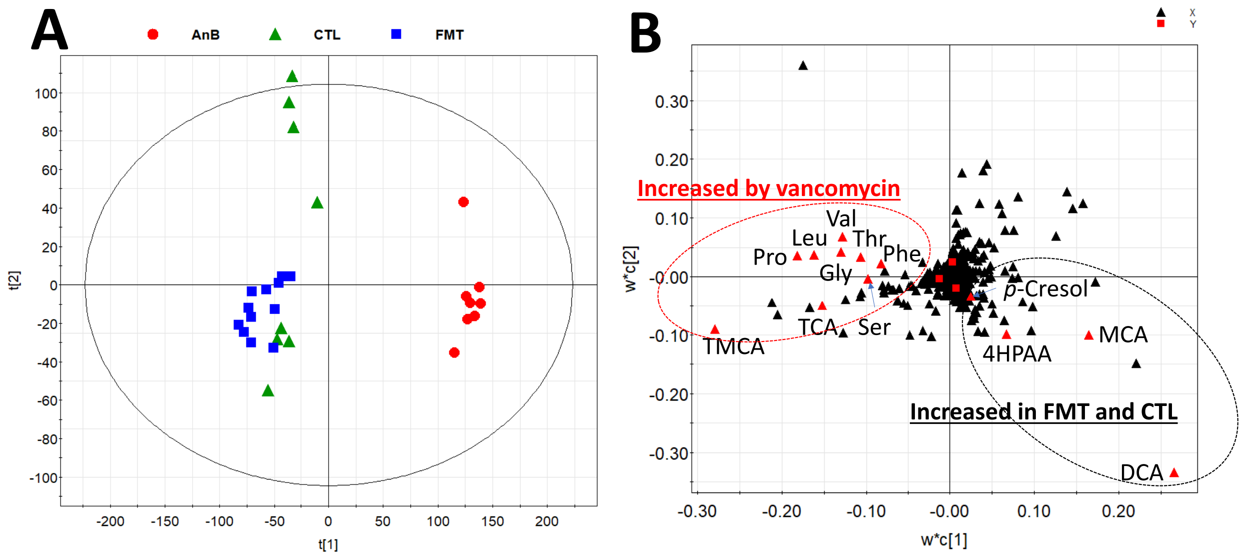

Figure S5. Identification and characterization of antibiotics (AnB)- and FMT-responsive metabolites in the colon digesta of mice. (A) The scores plot of a PCA model on colon digesta samples of control, AnB, and FMT samples. (B) The loadings plot of the PLS-DA model. Major AnB- and FMT-responsive metabolites are labeled.
